# A Cohort-Based Comparative Study of Three Minimally Invasive Apical Prolapse Surgeries: Sacropexy, Pectopexy, and Lateral Suspension

**DOI:** 10.3390/jcm14176073

**Published:** 2025-08-28

**Authors:** María Luisa Sánchez-Ferrer, Isabel Ñíguez-Sevilla, Vicente Luis Ruiz-Cotorruelo, Julián Jesús Arense-Gonzalo

**Affiliations:** 1 Department of Obstetrics & Gynecology, “Virgen de la Arrixaca” University Clinical Hospital, 30120 Murcia, Spain; ruiz_vic@icloud.com; 2 Biomedical Research Institute of Murcia (IMIB-Arrixaca), University Clinical Hospital “Virgen de la Arrixaca”, 30120 Murcia, Spain; julianjesus.arense@um.es; 3 Division of Preventive Medicine and Public Health, Department of Public Health Sciences, University of Murcia School of Medicine, 30120 Murcia, Spain

**Keywords:** laparoscopic, lateral suspension, pectopexy, sacropexy, apical pelvic organ prolapse

## Abstract

**Background**: Laparoscopic sacropexy (SP) is widely recognized as the gold standard for addressing apical pelvic organ prolapse. Nonetheless, alternative laparoscopic procedures, such as pectopexy (PP) and Dubuisson’s laparoscopic lateral suspension (LLS), have gained traction due to their relative technical simplicity. **Objective**: This study aims to assess both the preoperative characteristics and surgical outcomes in a cohort-based comparative study of three minimally invasive apical prolapse surgeries. **Methods**: We conducted a prospective, single-center study involving patients treated laparoscopically for apical prolapse. The surgical approaches compared include: sacropexy (SP); laparoscopic lateral suspension following Dubuisson’s technique (LLS), and pectopexy (PP). **Results**: A total of 180 patients underwent surgery: 115 with SP, 33 with LLS, and 32 with PP. While some differences were observed in patient profiles—such as a lower average BMI and more advanced prolapse stages (III and IV) in the SP group—the rates of surgical failure (evaluated through apical recurrence, need for reintervention, pessary use, and persistent symptoms) did not differ statistically between groups. In terms of anatomical outcomes, only the total vaginal length (TVL) was notably longer in the SP group. A clinically important finding was the substantially reduced operative time with the alternative methods, particularly LLS, which took less than half the duration required for SP, without any increase in intraoperative complication rates. **Conclusions**: Further research, particularly well-designed randomized multicenter trials, is essential to establish the relative efficacy of the alternative approaches (LLS and PP) compared with the current gold standard, sacropexy.

## 1. Introduction

Apical pelvic organ prolapse (POP) represents an important challenge in urogynecologic surgery. It is estimated that over 24% of adult women present with symptoms related to pelvic floor dysfunctions [1]. Various laparoscopic techniques have been developed to restore pelvic anatomy and function [1]. Laparoscopic sacropexy (SP) involves anchoring the uterus, cervix, or vaginal vault to the anterior longitudinal ligament of the sacrum using synthetic mesh, which reinforces both the anterior and posterior vaginal wall. SP remains the gold standard for apical prolapse repair due to its high anatomical success rates and long-term durability. However, its technical complexity and potential complications—such as presacral hemorrhage and defecatory dysfunction—have led to the development of alternative techniques [2].

Laparoscopic pectopexy (PP), introduced as a safer alternative, anchors the mesh to the iliopectineal (Cooper’s) ligaments, avoiding the presacral space [3]. A 2023 randomized controlled trial found that PP significantly reduced mesh fixation time (45.0 ± 11.3 min vs. 54.7 ± 9.3 min, *p* = 0.019) and showed comparable improvements in quality of life and sexual function scores (P-QOL, PISQ-12) to SP [4,5].

Laparoscopic lateral suspension (LLS), particularly the Dubuisson technique, offers a mesh-based suspension to the lateral abdominal wall. The mesh is attached to the uterus or vaginal apex, and its lateral arms are routed extraperitoneally beneath the peritoneum without anchoring them to fixed anatomical structures. This technique allows for a reduction in operative risks while maintaining effective apical support, thus avoiding “deep pelvic dissection” (dissection into the presacral space to anchor the mesh to the anterior longitudinal ligament of the sacrum—an area rich in critical vascular and neural structures such as the middle sacral vessels and hypogastric plexus) [6]. Both LLS and PP have been associated with shorter operative times and fewer complications, particularly in patients with higher surgical risk [4,7].

Despite these promising findings, direct comparative data remain limited. There are relatively few prospective, comparative, and randomized clinical trials evaluating surgical outcomes [8].

Additionally, anatomical correction does not always correlate well with functional improvement. Despite the fact that functional outcomes are the most relevant to patients’ quality of life, they remain underassessed in many studies.

The literature points to sacrocolpopexy as the reference procedure for its correction, although the quality of the evidence is moderate. Moreover, there is no consensus regarding its execution; it involves high complexity and requires a long learning curve, which hinders its implementation and has consequent repercussions for patients.

As a solution to these problems, simpler techniques such as pectopexy have emerged, supported by the available evidence as a good alternative, although further evaluation is still needed.

With the same objective, laparoscopic lateral suspension has appeared, which, although it has shown good preliminary results, has been evaluated in only a few clinical trials comparing it to the reference surgery. Furthermore, there is no study directly comparing the outcomes of pectopexy versus laparoscopic lateral suspension, so it is not yet clear which of the two could be considered the better alternative to the complex sacropexy.

Based on the above, the present study aims to compare, for the first time, the anatomical and functional outcomes among patients operated on using pectopexy, laparoscopic lateral suspension, and sacropexy, as well as to evaluate surgical times, intraoperative complications, and postoperative complications, in order to determine whether there are clinically important differences among the three techniques employed. This study aims to evaluate the perioperative outcomes, anatomical success, and patient-reported satisfaction among SP, PP, and LLS in the surgical management of apical POP. We hypothesize that pectopexy and laparoscopic lateral suspension achieve anatomical and functional outcomes comparable to sacrocolpopexy, with shorter operative times and fewer complications.

## 2. Materials and Methods

This is a single-center prospective study of patients undergoing laparoscopic repair of apical prolapse. The 3 techniques that we currently offer in our service were compared: sacropexy (SP), Dubuisson laparoscopic lateral suspension (LLS), and pectopexy (PP) groups.

The inclusion criteria were patients with primary or recurrent symptomatic prolapse in stage ≥ II according to the POP-Q. We excluded women with cervical elongation (defined as POP-Q Point C minus Point D ≥ 4). It was possible to perform hysteropexy, cervicopexy, or colpopexy in all groups. The exclusion criteria for hysteropexy (in these cases, we perform supracervical hysterectomy) were contraindications for uterine preservation: uterine pathology, risk of ovarian/tubal cancer (BRCA 1 and 2), or endometrium treatment with tamoxifen, and inability to follow a gynecologic cancer prevention program.

Other exclusion criteria were a history of open abdominal prolapse reconstructive surgery, a history of prolapse reconstructive surgery with vaginal mesh, stage I according to the POP-Q classification, asymptomatic prolapse, a medical contraindication for general anesthesia, and patient preference for treatment via vaginal surgery.

The primary outcome was treatment failure, which is defined as the existence of any of the following 2 elements:

(1) New treatment for prolapse (pessary placement or surgery);
(2) Anatomical outcomes, defined as recurrence of apical prolapse: stages ≥ II and any non-static POP-Q measurement greater than 0. Non-static “POP-Q measurements” refer to those obtained during a maximal Valsalva maneuver. We considered a measurement pathological when any point (Aa, Ba, C, Ap, Bp, or D) descended to ≥−1 cm relative to the hymenal plane, in line with ICS recommendations for defining stage II or greater prolapse.

For the primary analysis, this outcome is defined as any statistically significant difference (*p* < 0.05) in the following:

1. Subsequent therapeutic intervention, such as the need for a new treatment for prolapse (pessary placement or surgery);
2. Anatomical outcomes, defined as recurrence of apical prolapse (stage II or higher) and any non-static POP-Q measurement greater than 0.

The secondary objectives were to assess if there were differences in symptoms, measured using the validated PFDI-20 questionnaires (specifically, the following question: “Do you notice a sensation of lump in your genitals?”), including the analysis of the presence, severity, and impact of symptoms or discomfort derived from prolapse, urinary, and intestinal questionnaires (POPDI-6, CRAD-8, and UDI-6) and PISQ-12, as well as surgical times, complications, adverse events, and individual anatomical measurements in the POP-Q examination.

The surgical techniques can be seen in Supplementary Materials Annex 1.

The study was conducted in accordance with the Declaration of Helsinki and approved by the Ethics Committee of CEIm Hospital Virgen de la Arrixaca (protocol code 2022-3-8-HCUVA and date of approval: 26 April 2022). Written informed consent was obtained from all subjects involved in the study.

A pre-surgery visit and 3 follow-up visits (1 month, 6 months, and 1 year post-surgery) were planned. The study began in October 2022 and was finished in June 2024

Follow-up data were collected by a specialist who did not know which surgical technique was performed for each patient to eliminate the possibility of bias in the assessment of post-surgical results, including POP-Q measurements that were performed by blinded assessors to the type of surgery.

Statistical methodology: Null hypothesis (H_0_): Differences in anatomical success (POP-Q ≤ stage I), functional improvement, operative times, or complication rates were evaluated among patients undergoing sacrocolpopexy, pectopexy, or laparoscopic lateral suspension.

Alternative hypothesis (H_1_): The surgical techniques (pectopexy or laparoscopic lateral suspension vs. sacrocolpopexy) were evaluated to find differences in anatomical success, functional improvement, operative times, or complication rates. Quantitative variables were described using mean and standard deviation (SD); the Kruskal–Wallis test was used for their analysis. When statistically significant differences (*p* < 0.05) were found, the Mann–Whitney test was performed. Qualitative variables were described using percentages, and their analysis was carried out using Pearson’s Chi-square test. When statistically significant differences (*p* < 0.05) were found, a residual analysis was conducted. IBM^®^ SPSS^®^ software (v-25) was used for the statistical analysis; *p*-values < 0.05 were considered statistically significant.

## 3. Results

We operated on a total of 180 cases: 115 SP, 33 LLS, and 32 PP. There were no statistically significant differences (*p* < 0.05) in the mean age of the patients undergoing the three procedures. There were differences in BMI (body mass index): the mean was 27.51 (±4.45) kg/m^2^ for the PP group, and 25.67 (±3.69) kg/m^2^ for the SP group. On physical examination, both groups demonstrated comparable findings, although there were some differences in the baseline characteristics of the patients prior to surgery, such as anatomical differences, since the highest rates of apical stages III and IV were also higher in the SP. No statistically significant differences were identified between the groups in mode of delivery (vaginal or instrument-assisted), incidence of macroscopic fetuses, engagement in chronic physical exertion or sports, and prior vaginal surgeries. The LLS group had fewer previous hysterectomies than the other groups. Additionally, analysis of symptom-specific scales revealed no statistically significant (*p* < 0.05) differences between the groups in the mean scores of POPDI-6, CRAD-8, UDI-6, and PISQ-12 (Table 1).

Regarding surgical results, the highest rates of supracervical hysterectomies were performed in the SP group (78.3%) and the lowest in the LLS (6.1%) (*p* = 0.000). The surgical time was statistically longer in the SP [214.44 (±65.38) vs. LLS 108.79 (±34.93) and PP 163.83 (±49.80) minutes; *p* = 0.000]. No statistically significant differences were observed in the time required to perform the subtotal hysterectomy among the three groups. Consequently, the variations in total operative time between the three surgical techniques are not attributable to the hysterectomy itself, but rather to the duration associated with the specific prolapse repair method employed in each case. We also did not find clinically important differences in the rate of intraoperative and major postoperative complications (Table 2).

Although there were some differences in the baseline characteristics of the patients prior to surgery, such as a lower BMI for the SP group and anatomical differences, since the highest rates of apical stages III and IV were also in the SP, it is interesting to consider the other two alternative techniques because there were no statistically significant differences in the failure rate, measured by the apical recurrence rate, reintervention rate, or use of pessaries and symptoms. Interestingly, there were more recurrences in the posterior compartment in the LLS group. This could be due to the fact that the correction angle with this technique is more anterior, which (especially if overcorrected) could result in weakening of the posterior vaginal wall (Table 2).

Regarding the POP-Q measures, we only found differences in the higher TVL in the SP. Although these differences were statistically significant, they were not clinically relevant, as they were not perceived by the patients. However, the much shorter surgical time in alternative techniques is notable (LLS took less than half the time compared with the time used for SP). After surgery, the symptom scales (POPDI-6, CRAD-8, UDI-6, and PISQ-12) revealed no statistically significant differences between the three groups in the mean values (Table 2).

## 4. Discussion

To date, no studies have been identified in the medical literature that directly and simultaneously compare the three main minimally invasive techniques for apical prolapse repair—laparoscopic sacropexy, pectopexy, and laparoscopic lateral suspension (LLS)—within the same patient cohort. Available evidence is limited to pairwise comparisons between two of these techniques, and no clinical trial has yet evaluated all three in parallel [4,5,7,9]. Nevertheless, the existing evidence from randomized controlled trials, observational studies, systematic reviews, and meta-analyses supports the effectiveness of all three procedures in achieving high anatomical and subjective success rates, each with distinct surgical and functional profiles.

This comparative analysis of the three techniques provides relevant insights into the anatomical and functional outcomes of three abdominal approaches for apical prolapse repair.

Demographic and baseline characteristics of the patients were mostly homogeneous across groups, except for a higher BMI in the PP group and a greater prevalence of advanced apical prolapse stages (III–IV) in the SP group. These differences may reflect a selection bias, with SP reserved for more severe cases according with the data supporting in the literature about its superior long-term anatomical durability, lower recurrence rates, and reduced need for reoperation—especially in younger, sexually active women or those with recurrence risk factors (e.g., age < 60, stage III–IV prolapse, BMI > 26, shortened vaginal length, or prior failed repairs) [10]. However, in this study, these anatomical differences did not translate into clinically important disparities in subjective symptoms, as measured by validated scales (POPDI-6, CRAD-8, UDI-6, and PISQ-12), either before or after surgery.

Regarding surgical parameters in our study, SP was associated with significantly longer operative time compared to LLS and PP, despite similar durations for subtotal hysterectomies across groups. This finding supports previous research highlighting the technical complexity and longer learning curve of sacropexy [11]. In contrast, LLS required less than half the operative time of SP, positioning it as an efficient alternative for apical support, especially in high-risk or elderly patients [12,13,14]. Pectopexy also demonstrated favorable operative times [4,5,9] and may be considered an intermediate option between SP and LLS.

Laparoscopic sacropexy is associated with a higher risk of presacral hemorrhage, nerve or bowel injury [13,15]. Pectopexy has emerged as a safe and effective alternative, particularly in patients with contraindications to sacral dissection (such as obesity, pelvic adhesions, and vascular anomalies) or those at high risk for postoperative bowel dysfunction. By anchoring the vaginal apex to the iliopectineal ligaments and avoiding the sacral promontory. The main anatomical structures at risk are the external iliac vessels (lateral, more visible). Pectopexy offers shorter operative times, reduced blood loss, and a lower incidence of bowel dysfunction, without compromising anatomical outcomes. However, studies [4,5] have reported a higher rate of recurrent urinary symptoms postoperatively. Laparoscopic lateral suspension (LLS) is another effective option, especially for apical and anterior compartment prolapse. It offers benefits such as uterine preservation (although it is possible in sacropexy and pectopexy too), lower complication rates, and shorter surgical times, making it particularly suitable for obese patients or those with limited access to the sacral promontory [12,16]. Regarding postoperative complications, the only shared risk among the three techniques is mesh-related extrusion, such as exposure or erosion. However, the use of meshes is better than native tissues in terms of recurrence [1]. Sacropexy has a higher association with postoperative bowel issues, while pectopexy appears more prone to persistent or recurrent urinary symptoms [4]. LLS shows a favorable safety profile, though the evidence base is less robust compared to sacropexy [12]. Nevertheless, no statistically significant differences were found in intraoperative or major postoperative complications in this study.

Regarding efficacy, importantly, all three techniques yielded comparable rates of prolapse recurrence and reintervention, with no statistically significant differences in the need for pessaries or further surgery. Although SP showed slightly better apical correction on POP-Q point C, this was not associated with better symptom control or quality-of-life scores. This finding has been reported previously by other investigators [4,5,7]. An interesting finding was the significantly higher total vaginal length (TVL) achieved in the SP group. While a longer TVL may be considered an anatomical advantage, it did not correlate with improved patient-reported outcomes. The similar postoperative PISQ-12 scores across all groups suggest that sexual function was preserved regardless of the procedure. One notable difference was observed in posterior compartment recurrence, which was more frequent in the LLS group. This could be related to the lateral vector of suspension not adequately correcting posterior defects, a limitation previously described in the literature [12,13,16] because the efficacy of LLS may be reduced in cases of severe posterior compartment prolapse, and long-term data are less extensive than for sacropexy. In contrast, SP may provide better correction of multicompartment prolapse, as it involves more central fixation points. Pectopexy, although newer, demonstrated comparable outcomes to both LLS and SP in terms of apical support, operative safety, and functional recovery. However, its role in posterior compartment repair remains under investigation [5].

Therefore, the choice of surgical technique should be individualized based on patient-specific clinical and anatomical factors, including compartment involvement, desire for uterine preservation, baseline bowel or urinary function, surgical history, and patient preferences. Preoperative assessment tools—such as symptom questionnaires, urodynamic studies, and risk stratification protocols—are essential to reduce complications and optimize outcomes. In summary, although current studies suggest that all three techniques yield comparable short-term effectiveness and safety, there are important differences in perioperative characteristics, complication profiles, and ideal indications. The absence of studies directly comparing all three techniques within a single clinical trial highlights the need for future well-designed comparative research to establish evidence-based recommendations.

Our study’s main strength lies in its single-center, comparative design and relatively large sample size, especially for SP. We provided a comprehensive analysis, including POP-Q data, complications, recurrence, and validated symptom scales. However, limitations include variability in surgical indications, as well as the relatively shorter follow-up for LLS and PP, which may underestimate late recurrences.

Limitations: This is a prospective, single-center study in which the authors aim to compare real-world clinical outcomes in a setting where all three techniques described in the literature are available. The objective is not to conduct a clinical trial, but rather to present our experience with these techniques, applying the indications suggested by the literature—namely, considering sacropexy as the gold standard, but avoiding it in patients with constipation, obesity, or low back pain. For the two other alternative techniques, the differences in indication and outcomes remain unclear. Our data, despite the limitations inherent to a prospective, non-randomized design, suggest that LLS may provide better correction of the anterior and apical compartments due to the resulting vaginal angle, whereas the angle achieved in pectopexy might be more similar to that of sacropexy.

Regarding follow-up time, it indeed differs among techniques, as sacropexy was the first procedure we introduced, followed by pectopexy, and lastly LLS. Nonetheless, randomized clinical trials are needed to confirm these observations.

## 5. Conclusions

In conclusion, LLS and PP appear to be effective and less time-consuming alternatives to SP for apical prolapse repair, particularly in selected patients. SP remains a robust option for complex or multi-compartment prolapse, but its longer operative time and technical demands must be considered. Ongoing randomized studies with longer follow-up are needed to further define the indications and long-term outcomes of these evolving techniques, although it is questionable whether RCT is realistic given the many different variables involved (shorter operation time with a longer vagina), and numbers should be very high to evaluate complications.

## Figures and Tables

**Table 1:** Descriptive analysis of the laparoscopic surgery sample (n = 180).

Characteristics	Sacropexy (SP) (n = 115)	Dubuisson (LLS) (n = 33)	Pectopexy (PP) (n = 32)	*p*-Value
Age (years), mean (SD)	54.8 (10.1)	57.0 (7.6)	57.4 (9.9)	0.148
BMI (kg/m^2^), mean (SD)	25.7 (3.7)	26.8 (3.4)	27.5 (4.5)	0.025
Multiparous, n (%)	108 (94.0)	31 (94.0)	32 (100.0)	0.014
Vaginal births, n (%)	114 (99.1)	31 (94.0)	32 (100.0)	0.356
Previous hysterectomy, n (%)	25 (21.7)	2 (6.1)	9 (28.1)	0.049
Apical/uterine prolapse stage ≥ II				0.001
Stage II	31 (26.5)	19 (59.4)	18 (58.1)	
Stage III	65 (56.5)	12 (36.4)	9 (29.0)	
Stage IV	19 (16.5)	2 (6.1)	4 (12.9)	
Posterior prolapse ≥ II				0.356
Stage II	64 (55.7)	2 (6.1)	28 (87.5)	
Stage III	37 (32.2)	0 (0.0)	4 (12.5)	
Stage IV	14 (12.1)	0 (0.0)	0 (0.0)	
Constipation (preoperative), n (%)	11 (9.6)	5 (15.2)	9 (28.1)	0.026

BMI: body mass index; LLS: laparoscopic lateral suspension; SP: sacropexy; Constipation was a preoperative exclusion criterion for sacropexy; n: number.

**Table 2:** Surgical outcomes.

Surgical Outcomes	SP (n = 115)	LLS (n = 33)	PP (n = 32)	*p*-Value
Subtotal hysterectomy, n (%)	90 (78.3)	2 (6.1)	9 (28.1)	<0.001
Operative time (min), mean (SD)	214.4 (65.4)	108.8 (34.9)	163.8 (49.8)	<0.001
Intraoperative complications, n (%)	3 (2.6)	1 (3.0)	0 (0.0)	0.214
Surgical reintervention, n (%)	6 (5.2)	2 (6.1)	0 (0.0)	0.406
Posterior prolapse recurrence (cumulative incidence)	2/115 (1.7%)	6/33 (18.2%)	2/32 (6.3%)	0.019
TVL (post-op), mean (SD)	8.81 (1.37)	7.93 (1.15)	7.74 (1.18)	<0.001
Follow-up duration (months), median (IQR)	15 (9–22)	7 (5–11)	7 (5–10)	<0.001

LLS: laparoscopic lateral suspension; SP: sacropexy; PP: pectopexy; TVL: total vaginal length; IQR: interquartile range.

## Data Availability

All the data used to support the findings of this study are included in the article.

## Supplementary Materials

The following supporting information can be downloaded at: https://www.mdpi.com/article/10.3390/jcm14176073/s1.

## Abbreviations

The following abbreviations are used in this manuscript:

POP	Pelvic organ prolapse
SCL	Sacropexy (Laparoscopic)
LLS	Laparoscopic lateral suspension
POPQ	Pelvic organ prolapse quantification
PFDI-20	Pelvic Floor Distress Inventory Questionnaire-Short Form 20
POPDI-6	Pelvic Organ Prolapse Distress Inventory
CRAD-8	Colorectal–Anal Distress Inventory
UDI-6	Urinary Distress Inventory
PISQ-12	Pelvic Organ Prolapse/Urinary Incontinence Sexual Function Questionnaire
BMI	Body mass index
